# *Halimeda gracilis* as a bioactive resource: exploring its antioxidant, antibiofilm, anti-inflammatory, and antibacterial potential for dental applications

**DOI:** 10.2340/biid.v12.43612

**Published:** 2025-05-14

**Authors:** Dileepkumar Hemamalini, S. Shantha Sundari, K.M. Shahul Hameed Faizee, Sivakamavalli Jeyachandran

**Affiliations:** a Department of Orthodontics and Dentofacial Orthopedics, Saveetha Dental college, Saveetha Institute of medical and technical science (SIMATS)Saveetha University, Chennai, Tamilnadu, India; b Department of Orthodontics and Dentofacial Orthopedics, Sathayabama Dental college and Hospital, Sathyabama Institute of science and technology, Sathyabama University, Chennai, Tamilnadu, India; c Lab in Biotechnology and Biosignal Transduction, Department of Orthodontics, Saveetha Dental College and Hospital, Saveetha Institute of Medical and Technical Sciences (SIMATS), Saveetha University, Chennai, India

**Keywords:** *Halimeda gracilis*, bioactive polysaccharides, white spot lesions

## Abstract

**Aim:**

This study aimed to evaluate the antibacterial, antibiofilm, antioxidant and anti-inflammatory properties and of *Halimeda gracilis* extracts.

**Materials and methods:**

The *H. gracilis* sample was washed and extracted using methanol. The mixture was homogenized using a blender and centrifuged at high speed (10,000 × g) for 2 min, then stirred at room temperature for 30 min using magnetic stirrer, to ensure thorough extraction. Afterward, it was centrifuged at 5,000 × g for 10 min to separate the dissolved components from undissolved debris. Following this antioxidant activity was assessed using DPHH assay, the antimicrobial effects were tested against *Streptococcus mutans*, *Escherichia coli*, *Enterococcus faecalis* and *Shigella sonnei* using Kirby-Bauer disk diffusion, biofilm inhibition assay was done to assess biofilm inhibition against *S. mutans*, *E. coli*, *E. faecalis* and *S. sonnei*. Finally, the anti-inflammatory activities of the *H. gracilis* were determined using a modified version of the BSA assay.

**Results:**

When tested against *S. mutans*, *E. coli*, *E. faecalis*, and *S. sonnei* strains, the antimicrobial evaluation revealed that the extract successfully inhibited biofilm formation when tested against the same organism, and it also demonstrated increased activity with increasing concentration. The zone of inhibition progressively expanded with increasing concentration, reaching a maximum of 17 mm ± 0.1 for 100 µg/mL. In terms of antioxidant activity, the *H. gracilis* metholic extract gradually increased from 10 µg/mL to a higher activity at 40 µg/mL in comparison to the control and blank, and then decreased at a dose of 50 µg/mL. At different doses, the anti-inflammatory action of *H. gracilis* extracts successfully inhibited BSA denaturation, which causes inflammation; the maximum activity has been observed.

**Conclusion:**

This comprehensive analysis highlights *H. gracilis* as a valuable natural resource with multifaceted biological activities, supporting its further investigation for therapeutic applications in dentistry.

## Introduction

*Halimeda gracilis* is a species of green macroalgae commonly found in tropical and subtropical marine environments. It is characterized by its segmented, calcified structure, which contributes to the formation of coral reef ecosystems [1]. This calcified structure, rich in calcium carbonate, has gained interest in dentistry for its potential use in bone regeneration and biomaterials [2].

The algae’s high mineral content and biocompatibility make it a promising candidate for creating scaffolds and fillers in bone grafts or tooth repair. Research suggests that its natural composition could support the regeneration of bone tissue, particularly in dental implants and periodontal therapies [3].

The bioactive components found in seaweeds possess antibacterial, anti-inflammatory, antioxidant and anticancer properties [4]. The bioactive components have also demonstrated encouraging outcomes in the prevention and treatment of oral disorders, such as dental caries and biofilm formation. Species such as *H. gracilis Ulva* ***l****actuca, Padina, Sargassum, Gracilaria* have been used in dentistry because they have been shown to have antibacterial activity against oral bacteria, may help reduce inflammation, and can lower the amount of germs and plaque in the mouth. These compounds offer a sustainable and biocompatible approach to enhancing oral health care practices [5–9].

Demineralization/dissolution of tooth hard structures, such as cementum, dentin, and enamel**,** is inevitable due to various environmental and genetic factors. Enamel is at the forefront of dental demineralization for anatomical reasons; from a homeostasis perspective, partial enamel dissolution is essential to restoring the equilibrium of mineral content (i.e., calcium phosphates) in oral fluids [10, 11]. Fluoride-based formulations have been routinely used for the past decades to control the mineral imbalance between the environment and mineralized tooth hard tissues, hence slowing the advancement of dental demineralization. Fluorides have not been shown to have any systemic negative effects, but their usage as the main epidemiological strategy to prevent dental caries is under scrutiny globally [12–14]. Many European Union member states, as well as a few US and Canadian municipalities, have implemented stringent regulatory measures or encouraged public opinion surveys about the consumption of fluorides through community water fluoridation in recent years [15].

The abundance of opinions supporting and opposing the intentional and ongoing administration of fluorides demonstrates a rhetorical conflict that is far from being settled. However, contentious competition like this also presents a rare chance to look for medicinal substitutes that can deliver more efficient action.

The goal of this study was to investigate the potential of the calcareous macroalga *H. gracilis* by evaluating its antibacterial, anti-inflammatory, antioxidant, and antibiofilm properties, as well as its potential use in dentistry as an enamel-mineralizing agent due to its high calcium content.

## Materials and methods

### Sample collection and preparation

**Figure 1:**
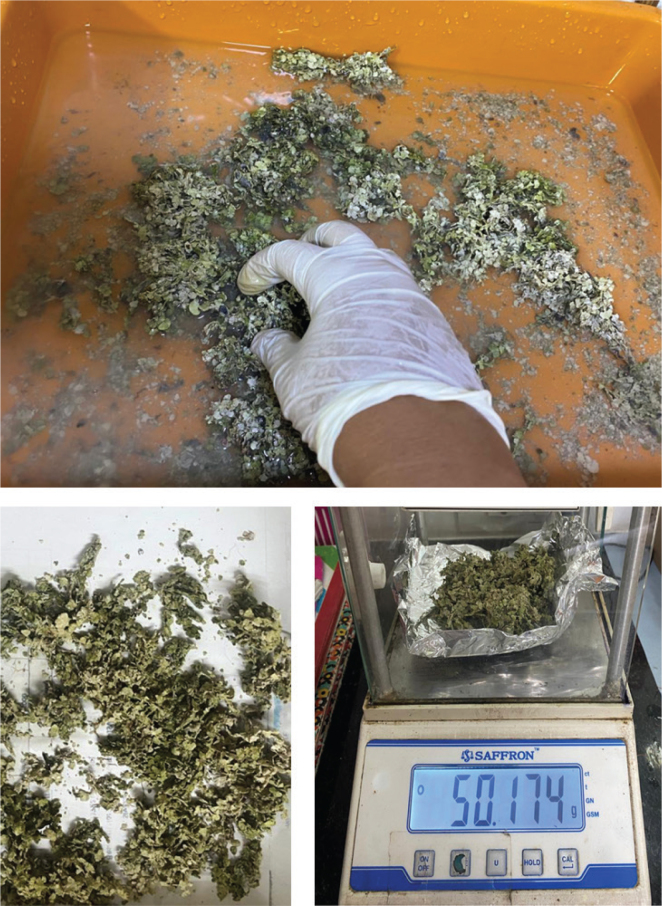
Processing, drying and fine powdering of Halimeda gracilis.

The sample, which was collected from several locations in Rameshwaram, Tamilnadu, India was determined to be *H. gracilis.* The sample was cleaned in seawater to remove any debris (Figure 1) and allowed to dry for 2 days in the sun. It was then rinsed with tap water to remove any remaining sand and salt and allowed to dry in the sun for 7 days before being finely chopped with a scalpel to create a powder.

### Solvent extract

The *H. gracilis* powder (40 g) was mixed with three volumes of methanol following the method of Terada et al. [16]. The mixtures were homogenized at 10,000 × g for 2 min in a homogenizer. The homogenate was then stirred continuously at room temperature for 30 min. The mixtures were centrifuged at 5,000×g for 10 min at room temperature using a cooling centrifuge REMI (C-24 Plus, Goregaon [East], Mumbai, Maharashtra, India) to remove undissolved debris.

### Antibacterial activity

The antibacterial efficacy of the *H. gracilis* extract was assessed using the Kirby-Bauer method [17] *Streptococcus mutans* (Sdc_ortho5 16S Ribosomal RNA gene, partial sequence GenBank:OQ947767.1), *Enterococcus faecalis* (NCT34 16S Ribosomal RNA gene, partial sequence GenBank:OM283553.1), *Shigella sonnei* (NCT34 16S Ribosomal RNA gene, partial sequence GenBank OM283552.1) and *Escherichia coli (16S Ribosomal RNA gene, partial sequence, GenBank: U00096.3)* were the bacteria chosen for accessing the antibacterial activity. The bacterial inoculum was grown in nutrient broth overnight and a fixed volume was inoculated into 10 mL aliquots nutrient agar, mixed and then poured over a nutrient agar base in sterile petridishes; this formed the bacterial lawn. The surfaces of the plates were then inoculated with 200 μL of bacterial suspension. Following the well diffusion technique, wells loaded with 25, 50, 75, and 100 μL of *H. gracilis* were placed onto agar plates. Each Petri plate was individually sealed to prevent potential contamination and media evaporation. The plates were promptly incubated for 24–48 h at 37°C. The antibacterial effects were indicated by the clear zone of inhibition surrounding the wells, and the diameter of each inhibition zone was measured (in mm) by vernier caliper (Baker Gauges India Pvt. Ltd. – Pune, Maharashtra).

### Biofilm inhibition assay

Biofilm production and development were studied using light microscopy following Crystal Violet staining. Following the protocol previously described by Zhou [18] biofilms were cultured in the wells of a 24-well microtiter plate and stained with Crystal Violet stain (Hi-media, India). Freshly cultured bacterial suspension of *S. mutans*, *E. coli, E. faecalis* and *S. sonnei* was added to each well of a 24-well polystyrene microtiter plate (Corning, Mumbai, India), which had previously been lined with sterile glass cover slips for microscopic examination. The plates underwent additional incubation, and after 48 to 72 h, the cover slips were gathered. Glass squares measuring 1 × 1 cm were utilized to investigate the potential inhibitory properties of *H. gracilis* extracts on in vitro biofilm formation. Biofilms were allowed to develop on these glass squares placed in 24-well polystyrene plates containing *H. gracilis* extracts (25 µg – 100 µg/mL) and were incubated for 24 h at 30°C. A sterile medium without bacteria was used as control. Subsequently, the glass squares were retrieved and rinsed twice with Phosphate Buffer solution (PBS) before microscopy. Staining was performed using Crystal Violet (6%) stain, and qualitative examination was carried out using an Olympus CX21i LED Microscope, (Olympus Corporation, Tokyo, Japan) at 40x magnification.

### Antioxidant activity - DPPH radical scavenging activity

The DPPH’ (2,2-Diphenyl-1-picrylhyrazyl) radical scavenging activity was calculated using the technique described by Sushant Shekar [19]. 10 mL of methanol was used to dissolve 10 mg of *H. gracilis.* 24 mg of DPPH (Sisco Research Laboratories Pvt. Ltd. [SRL] – India) was dissolved in 100 mL methanol to create the stock solution, which was then stored in the refrigerator until needed. The DPPH stock solution was diluted with methanol to yield the working solution, which had an absorbance of around 0.98 (±0.02) at 517 nm. After mixing 3 mL of the working solution with 100 μL of either a standard solution (positive control) consisting of ascorbic acid or the extract in a glass vial, the absorbance was measured using a Shimadzu Spectroph-otometer (Shimadzu UV-1900i Spectrophotometer, Shimadzu Corporation, Kyoto, Japan) at 517 nm for 30 min. PBS was used as blank negative control**.** The scavenging activity percentage was computed for concentrations of *H. gracilis* of 10, 20, 30, 40 and 50 µg/mL. The concentration of DPPH following reaction with an antioxidant sample at time *t* is given by [DPPH]T = *t*. Duplicates were made of the prepared solution

**Radical scavenging activity** *%RSA = (control-sample/control)*100

Where:

**Control** = Absorbance of the blank

**Sample** = Absorbance of the test sample (with antioxidant).

### Anti-inflammatory activity – Bovine serum albumin assay

The anti-inflammatory activity of the *H. gracilis* was determined using a modified version of the BSA assay reported by Williams et al. [20]. BSA solution (0.4%, w/v) was prepared in Tris Buffered Saline (one tablet was dissolved in 15 mL of deionized water to yield 0.05 M Tris and 0.15 M sodium chloride, pH 7.6 at 25°C). The pH was adjusted to 6.4 with diluted glacial acetic acid. Stock solutions of *H. gracilis* were prepared in methanol at a concentration of 50 μg/mL or 0.005%, w/v. Respective aliquots of 5.0, 10 and 20 µL representing concentrations of 0.25, 0.50 and 1.00 µg/mL of the stock solutions were added to test tubes containing 1 mL of 0.4%, w/v BSA buffer solution (prepared by dissolving 1 g BSA in 80 mL PBS and adding distilled water to obtain 100 mL). A negative (Dimethyl sulfoxide [DMSO]) and a positive (Aspirin) control were assayed in a similar manner. The solutions were then heated in a water bath at 72°C for 10 min and cooled for 20 min under laboratory conditions. The turbidity of the solutions (level of protein precipitation) was measured at 660 nm in a Shimadzu Spectrophotometer (UV-1900i, Shimadzu Corporation, Tokyo, Japan) using a water blank. The experiments were conducted in duplicate and the mean absorbance values were recorded. The percentage inhibition of precipitation (protein denaturation) was determined on a percentage basis, relative to the negative control using the following equation:

*%Inhibition = Abs of standard – Abs of sample/Abs of standard*100

### Statistical analysis

The results were analyzed by one way ANOVA and in case of significant differences by post hoc test (Tukey’s Honest Significant Difference [HSD]). The statistical analyses were performed using SPSS version 25 and the level of significance was set at *p* = 0.05.

## Results

### Antibacterial activity

**Figure 2:**
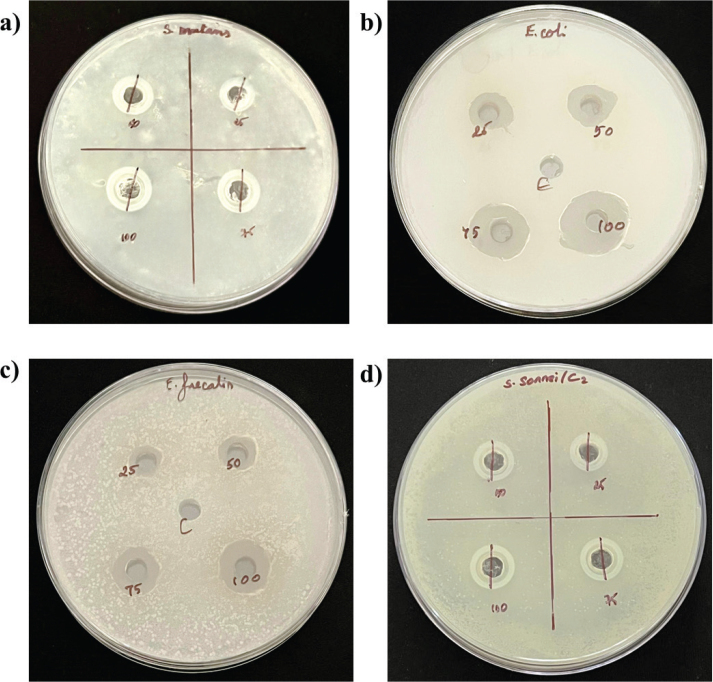
Antibacterial activity of Halimeda gracilis against (A) Streptococcus mutans (B) Escherichia coli (C) Enterococcus faecalis and (D) Shigella sonnei.

**Figure 3:**
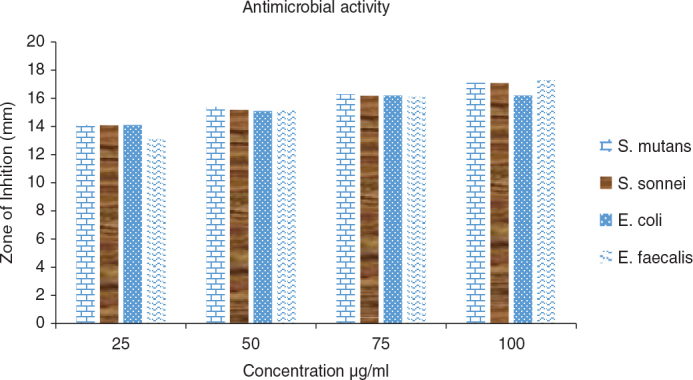
Antibacterial activity (Zone of inhibition) of *Halimeda gracilis* at varied concentration.

The antibacterial activity of the tested samples was evaluated against *E. faecalis, E. coli, S. sonnei, and S. mutans* at different concentrations (25, 50, 75, and 100 µg/mL). The mean inhibition zone diameters (mm) and standard deviations are presented in Table 1 and Figures 2 and Figure 3.

**Table 1:** Antibacterial response (diameter of inhibition zone ) of four different concentration of Halimeda gracilis against the four bacterial groups.

Concentrations µg/mL	Groups	Mean (mm)	Standard deviation (mm)	*p*
**@25**	***Enterococcus faecalis***	13.100	0.1000	0.000*
	***Escherichia coli***	14.133	0.1528	
	***Shigella sonnei***	14.200	0.1000	
	***Streptococcus mutans***	14.100	0.1000	
**@50**	***Enterococcus faecalis***	15.133	1.1015	0.947
	***Escherichia coli***	15.067	1.0504	
	***Shigella sonnei***	15.433	0.4933	
	***Streptococcus mutans***	15.233	0.2082	
**@75**	***Enterococcus faecalis***	15.133	1.7616	0.643
	***Escherichia coli***	15.867	0.5774	
	***Shigella sonnei***	15.600	1.2166	
	***Streptococcus mutans***	16.300	0.0000	
**@100**	***Enterococcus faecalis***	17.300	0.0000	0.315
	***Escherichia coli***	15.867	0.5774	
	***Shigella sonnei***	16.767	0.5774	
	***Streptococcus mutans***	16.100	1.7321	

While significant differences were observed among the four bacterial groups at a *H. gracilis* concentration of 25 µg/mL (*p* = 0.000), no statistically significant differences (*p* > 0.05) were detected at higher concentrations of *H. gracilis* (50, 75, and 100 µg/mL). At 25 µg/mL, post hoc analysis revealed that *E. faecalis* exhibited a significantly smaller inhibition zone compared to *E. coli*, *S. sonnei*, and *S. mutans* (Table 2).

**Table 2:** Post hoc analysis of antibacterial responses between the four bacterial groups at a concentration of Halimeda gracilis of 25 µg/mL.

Concentr ation µg/mL	Intragroup comparison		Mean difference	Standard error	Sig.
**@25**	***Enterococcus faecalis***	***Escherichia coli***	-1.0333	0.0943	0.000*
		***Shigella sonnei***	-1.1000	0.0943	0.000*
		***Streptococcus mutans***	-1.0000	0.0943	0.000*
	***Escherichia coli***	***Shigella sonnei***	-0.0667	0.0943	0.892
	***Shigella sonnei***	***Streptococcus mutans***	0.0333	0.0943	0.984
		***Streptococcus mutans***	0.1000	0.0943	0.721

### Antibiofilm activity

**Figure 4:**
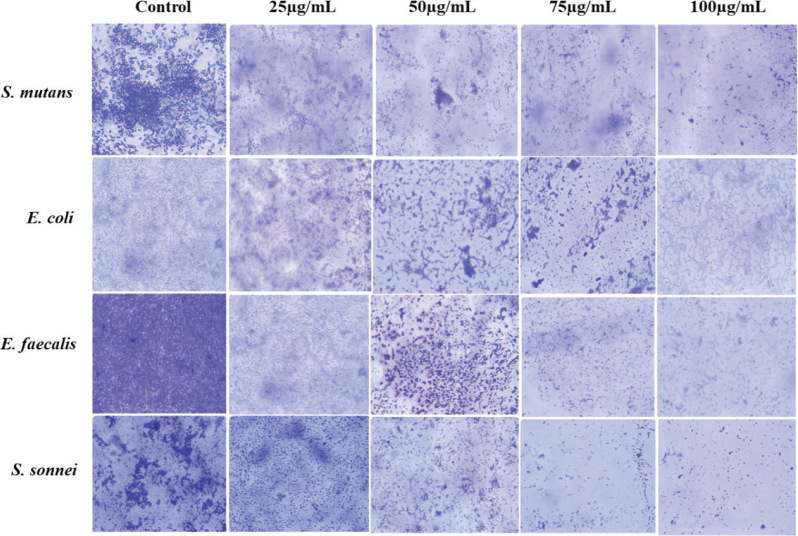
Light microscopic images showing antibiofilm activity at different concentrations of Halimeda gracilis against (A) Streptococcus mutans (B) Escherichia coli (C) Enterococcus faecalis (D) Shigella sonnei.

The extract of *H. gracilis* was evaluated for antibiofilm activity against *S. mutans*, *E. coli*, *E. faecalis*, and *S. sonnei* and the results are presented in Figure 4. The control group showed dense bacterial growth across all four bacterial species, indicating normal proliferation in the absence of *H. gracilis* extract. At a concentration of 25 µg/mL, noticeable reductions in bacterial density were observed, although clusters remained visible, suggesting partial inhibition. At 50 µg/mL, there was increased bacterial dispersal with fewer clusters, and cell death was evident. At 75 µg/mL, further reduction in bacterial count was observed, with only a few visible colonies, indicating strong antimicrobial activity. Finally, at 100 µg/mL, minimal bacterial presence was detected, with only a few scattered cells, suggesting near-complete inhibition.

## Antioxidant activity

**Figure 5:**
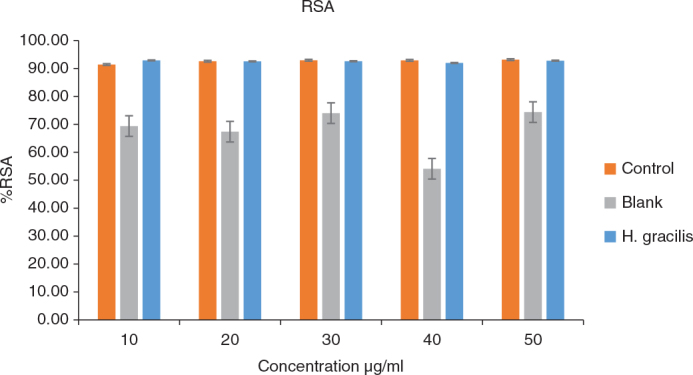
Antioxidant activity (% Radical scavenging activity) of Halimeda gracilis at varied concentration.

The antioxidant response of *H. gracilis* was evaluated at five different concentrations (10, 20, 30, 40, and 50 µg/mL) and compared to a blank and control group (Table 3 and Figure 5). For each of the five *H. gracilis* concentrations, there were significant differences between the blank, control, and *H. gracilis* groups (*p* = 0.000). While there were no significant differences between the control and *H. gracilis* groups, the post hoc tests revealed that the absorbance of the blank group was considerably higher than that of both groups at all *H. gracilis* concentrations (Table 4), indicating lower radical scavenging activity.

**Table 3:** Antioxidant response (measured in nm) for five different concentration of Halimeda gracilis.

Concentration µg/mL	Groups	Mean (nm)	Standard deviation nm	*p*
**@10**	**Blank**	0.26700	0.008185	0.000*
	**Control**	0.05433	0.004509	
	***H. gracilis***	0.04133	0.002309	
**@20**	**Blank**	0.26700	0.008185	0.000*
	**Control**	0.05433	0.004509	
	***H. gracilis***	0.04367	0.003512	
**@30**	**Blank**	0.26700	0.008185	0.000*
	**Control**	0.05433	0.004509	
	***H. gracilis***	0.04333	0.005774	
**@40**	**Blank**	0.26700	0.008185	0.000*
	**Control**	0.05433	0.004509	
	***H. gracilis***	0.05167	0.002309	
**@50**	**Blank**	0.26700	0.008185	0.000*
	**Control**	0.04000	0.001732	
	***H. gracilis***	0.04233	0.002309	

**Table 4:** Post hoc analysis on antioxidant responses between three groups over different concentrations of Halimeda gracilis.

Concentration (µg/mL)	Intragroup comparison		Mean difference in nm	Standard error	Sig.
**@10**	**Blank**	**Control**	0.212667*	0.004538	0.000*
		***H. gracilis***	0.225667*	0.004538	0.000*
	**Control**	***H. gracilis***	0.013000	0.004538	0.064
**@20**	**Blank**	**Control**	0.212667*	0.004706	0.000*
		***H. gracilis***	0.223333*	0.004706	0.000*
	**Control**	***H. gracilis***	0.010667	0.004706	0.138
**@30**	**Blank**	**Control**	0.212667*	0.005178	0.000*
		***H. gracilis***	0.223667*	0.005178	0.000*
	**Control**	***H. gracilis***	0.011000	0.005178	0.165
**@40**	**Blank**	**Control**	0.212667*	0.004538	0.000*
		***H. gracilis***	0.215333*	0.004538	0.000*
	**Control**	***H. gracilis***	0.002667	0.004538	0.832
**@50**	**Blank**	**Control**	0.227000*	0.004092	0.000*
		***H. gracilis***	0.224667*	0.004092	0.000*
	**Control**	***H. gracilis***	-0.002333	0.004092	0.840

### Antiinflammatory

**Figure 6:**
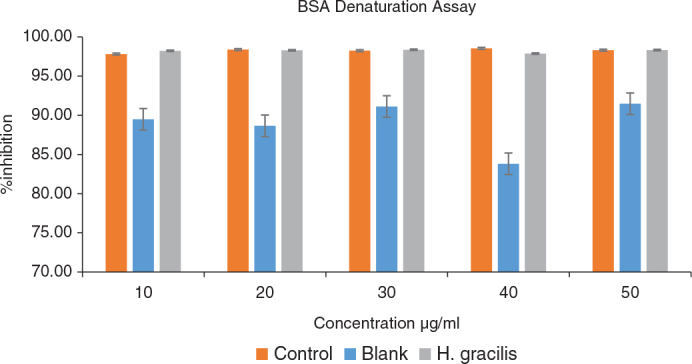
Anti-inflammatory activity (% Bovine serum albumin denaturation) of Halimeda gracilis at varied concentration.

The anti-inflammatory response of *H. gracilis* was tested at five different concentrations (10, 20, 30, 40, and 50 µg/mL) and compared to the blank and control groups (Table 5 and Figure 6). Only at a 50 µg/mL concentration of *H. gracilis* did the ANOVA reveal significant differences between the blank, control, and *H. gracilis* groups (*p* = 0.000). There were no significant differences between the control and *H. gracilis* groups at this concentration, but the post hoc tests revealed that the blank group had significantly higher absorbance and, hence, a lesser anti-inflammatory impact than the control and the *H. gracilis* groups (Table 6).

**Table 5:** Anti-inflammatory response for five different concentrations of Halimeda gracilis.

Concentration (µg/mL)	Groups	Mean (nm)	Standard deviation (nm)	*P*
**@10**	**Blank**	0.18467	0.142620	0.143
	**Control**	0.05367	0.003512	
	***H. gracilis***	0.04333	0.003055	
**@20**	**Blank**	0.18467	0.142620	0.142
	**Control**	0.05367	0.003512	
	***H. gracilis***	0.04333	0.000577	
**@30**	**Blank**	0.18467	0.142620	0.139
	**Control**	0.05367	0.003512	
	***H. gracilis***	0.04100	0.001732	
**@40**	**Blank**	0.18467	0.142620	0.161
	**Control**	0.05367	0.003512	
	***H. gracilis***	0.05433	0.001155	
**@50**	**Blank**	0.21733	0.002887	0.000*
	**Control**	0.04367	0.002309	
	***H. gracilis***	0.04300	0.001732	

**Table 6:** Post hoc analysis on anti-inflammatory responses between three groups at a concentration of Halimeda gracilis at 50 µg/mL.

Concentration (µg/mL)	Intragroup comparison		Mean difference	Standard error	Sig.
**@50**	**Blank**	**Control**	0.173667*	0.001925	0.000*
		***H. gracilis***	0.174333*	0.001925	0.000*
	**Control**	***H. gracilis***	0.000667	0.001925	0.937

## Discussion

*Halimeda gracilis,* a marine calcareous algae, exhibits diverse biological activities due to its phytochemical richness, including tannins, phenols, alkaloids, and terpenoids [1]. It has shown potential as an antioxidant, antidiabetic [5] antibacterial, anticancer [4] and spermicidal agent [6]. In bone and tooth repair, its high calcium carbonate content can be processed into coralline hydroxyapatite or hybrid hydroxyapatite/calcium carbonate composites. These materials mimic the natural structure of bone and tooth, promoting osteoconduction (supporting bone growth), osteoinduction (stimulating bone formation), and tissue integration [3]. Such compounds are particularly promising for enamel and dentin repair due to their biocompatibility and remineralization potential. In this study, we assessed the antibacterial, antibiofilm, antioxidant and anti-inflammatory activities of one such species *H. gracilis* to determine its potential application in dentistry.

Antibacterial activity was assessed for *H. gracilis* against *S. mutans*, *E. coli, E. faecalis* and *S. sonnei using* the Kirby-Bauer disc diffusion (KBDD) method since it is more economical and standardized one used for testing susceptibility to most of the antibiotics [17].

The antimicrobial activity of *H. gracilis* extract exhibited varying degrees of inhibition across bacterial species, with significant differences observed at lower concentrations. At 25 µg/mL, *E. faecalis* showed a significantly lower inhibition zone compared to *E. coli, S. sonnei,* and *S. mutans*, indicating reduced susceptibility to the extract at this concentration. However, at higher concentrations (50, 75, and 100 µg/mL), the inhibition zones increased, and no statistically significant differences were noted among the bacterial species, suggesting a more uniform antimicrobial effect across all tested bacteria.

The significant variation at lower concentrations may be due to intrinsic differences in bacterial cell wall composition and defense mechanisms, with *E. faecalis* possibly exhibiting higher resistance to the extract. The presence of bioactive compounds in *H. gracilis,* such as terpenoids, alkaloids, fatty acids, polysaccharides, and phenolic compounds, likely contributes to its antimicrobial potential against a broad spectrum of pathogens, including bacteria, fungi, and viruses. These compounds are known for their ability to disrupt microbial cell membranes, inhibit essential enzymes, and interfere with bacterial metabolic processes.

Supporting these findings, a study by Bashir et al. reported that *H. gracilis* was effective against *E. coli* but ineffective against *Staphylococcus aureus* [8], highlighting species-specific variations in susceptibility. Overall, the results indicate that *H. gracilis* possesses strong antibacterial properties, particularly at higher concentrations, making it a promising candidate for natural antimicrobial applications.

Using light microscopy following Crystal Violet staining, the biofilm inhibition of *H. gracilis* against *S. mutans, E. coli, E. faecalis*, and *S. sonnei* was assessed. In the current investigation, the antibiofilm activity of *H. gracilis* demonstrated a rise in biofilm inhibition when concentration increased from 25 to 100 µg/mL. The study of Suganya et al. on the anti-biofilm efficacy of marine algae *S. wightii* and *H. gracilis* showed significant antibiofilm formation activity up to 40–75% against gram-negative bacteria *(E. coli, P. aeruginosa, and V. parahaemolyticus)*, which corroborate our findings [19].

The antioxidant activity of *H. gracilis* was assessed at different concentrations, showing significant variation compared to the blank and control groups. The blank exhibited the highest antioxidant response at all concentrations, while *H. gracilis* showed a concentration-dependent effect. No significant difference was observed between *H. gracilis* and the control at 40 and 50 µg/mL, suggesting comparable activity at higher concentrations and a moderate antioxidant potential, likely due to bioactive compounds such as phenolics and flavonoids.

A study by Nazarudin et al. found that *H. opuntia e*xhibited DPPH reduction of 56.29–63.91%, achieving 50% inhibition at a concentration of 200 µg/mL, highlighting the antioxidant properties of Halimeda species. These findings suggest that *H. gracilis* may have potential applications in nutraceuticals, though further studies are needed to explore its therapeutic benefits [21].

The antiinflammatory activity of *H. gracilis* was assessed at various concentrations, revealing a notable response at higher concentrations. At 10–40 µg/mL, the blank exhibited the highest response, but no significant differences were observed between *H. gracilis* and the control. However, at 50 µg/mL, both the Control and *H. gracilis* groups showed significantly higher anti-inflammatory responses compared to the Blank group while no significant difference was observed between the Control and *H. gracilis* groups.

These findings align with previous studies on Halimeda species. Khorshidi et al. and Azim et al. reported that *H. opuntia* ethanol extract significantly inhibited paw edema in mice, particularly in later inflammation stages, likely by modulating prostaglandins. Similarly, *H. tuna* polysaccharides demon-strated anti-inflammatory and immunomodulatory effects by reducing inflammatory mediators such as nitric oxide and TNF-α in macrophage cells. These results suggest that bio-active compounds in Halimeda species may have therapeutic potential for managing inflam-mation, warranting further investigation [22, 23].

The presence of calcium and the abundance of bioactive substances such terpenoids, sterols, fatty acids, and poly-saccharides are two distinctive characteristics of *H. gracilis.* These substances are a possible source for the development of natural antibiotics because they have shown a variety of pharmacological actions, such as antibacterial and antifungal properties. Their potential for use in dentistry stems from the presence of calcium carbonate. Although this study was in vitro assessment precluding any conclusion on the in vivo application in dentistry of *H. gracilis,* the encouraging findings motivate investigating the remineralizing ability of *H. gracilis* to support its usage in dentistry.

## Conclusion

The study highlights the substantial therapeutic potential of *H. gracilis*, demonstrating significant antimicrobial, antibiofilm, antioxidant, and antiinflammatory effects. Its rich phenolic and flavonoid content suggests effective neutralization of free radicals, aiding in the prevention of oxidative stress-related diseases. The species shows promise as a natural antimicrobial agent, particularly for oral health, and its antiinflammatory and antibiofilm activities make it valuable for treating inflammation-related disorders and chronic infections. These findings support *H. gracilis* as a multi-functional bioactive resource for pharmaceuticals, cosmetics, and healthcare, warranting further research for therapeutic applications.
